# A randomised, double-blinded, placebo-controlled clinical study on intra-articular hyaluronan treatment in equine lameness originating from the metacarpophalangeal joint

**DOI:** 10.1186/s12917-016-0687-7

**Published:** 2016-03-23

**Authors:** Tytti M. Niemelä, Riitta-Mari Tulamo, Anna K. Hielm-Björkman

**Affiliations:** Department of Equine and Small Animal Medicine, Faculty of Veterinary Medicine, University of Helsinki,, P.O. Box 57, 00014 Helsinki, Finland

**Keywords:** Double-blinded, Clinical study, Lameness, Metacarpophalangeal joint, Non-animal stabilized hyaluronic acid (NASHA), Placebo-controlled

## Abstract

**Background:**

Intra-articular inflammation resulting in lameness is a common health problem in horses. Exogenous intra-articular hyaluronic acid has been shown to provide an analgesic effect and reduce pain in equine and human osteoarthritis. High molecular weight non-animal stabilized hyaluronic acid (NASHA) has gained popularity in the treatment of human arthritic conditions due to its long-acting pain-relieving effects. The aim of this study was to compare the response to treatment of lameness localized in the equine metacarpophalangeal joint injected with non-animal stabilized hyaluronic acid (NASHA) and placebo (saline). Twenty-seven clinically lame horses with a positive response to diagnostic intra-articular anaesthesia of the metacarpophalangeal joint and with no, or at most mild, radiographic changes in this joint were included in the study. Horses in the treatment group (*n =* 14) received 3 mL of a NASHA product intra-articularly, and those in the placebo group (*n =* 13) received an equivalent volume of sterile 0.9 % saline solution.

**Results:**

The change in the lameness score did not significantly differ between NASHA and placebo groups (*P =* 0.94). Scores in the flexion test improved more in the NASHA group compared with placebo (*P =* 0.01). The changes in effusion and pain in flexion were similar (*P =* 0.94 and *P =* 0.27, respectively) when NASHA and placebo groups were compared. A telephone interview follow-up of the owners three months post-treatment revealed that 14 of the 21 horses (67 %) were able to perform at their previous level of exercise.

**Conclusions:**

In the present study, a single IA NASHA injection was not better than a single saline injection for reducing lameness in horses with synovitis or mild osteoarthritis. However, the results of this study indicate that IA NASHA may have some beneficial effects in modifying mild clinical signs but more research is needed to evaluate whether the positive effect documented ie. reduced response in the flexion test is a true treatment effect.

## Background

Over 70 % of racehorses suffer from lameness during their lifetime, and the cause of this is often intra-articular inflammation [1]. Acute or repetitive overload injuries of the metacarpo- or metatarsophalangeal joint can lead to synovitis or capsulitis of the joint [2]. Damage to the articular cartilage, subchondral bone, synovium, joint capsule or other soft tissues of the joint may lead to degeneration [3]. Inflammation of articular soft tissues can result in the synthesis of defective cartilage matrix components, leading to osteoarthritis [4]. This may remain clinically silent for a long period and only become clinically and radiographically evident when the disease has progressed to an irreversible state [5, 6]. Therefore, early detection and treatment of the disease, as well as prevention of further damage, is considered important.

Hyaluronan (HA) is a glycosaminoglycan that has an important role in the formation of proteoglycan aggregates in the cartilage. It is also a component of the synovial fluid. Synovial membrane type B cells and chondrocytes synthetize HA by disaccharide oligomerization [7]. Hyaluronan molecules restrict large plasma proteins from entering the synovial fluid, but facilitate the passage of small molecules for the maintenance of nutrition [8]. In osteoarthritis, the synthesis of HA is disrupted by increased levels of pro-inflammatory cytokines, free radicals and proteinases, resulting in HA with a significantly reduced molecular weight, more molecular polydisaccharides and a reduction in synovial fluid viscoelasticity [9].

Exogenous intra-articular (IA) HA has been shown to provide an analgesic effect and reduce pain in equine [10–15] and human osteoarthritis [16–24]. It is believed that exogenous HA stimulates the synthesis of HA by synoviocytes and promotes proteoglycan synthesis by chondrocytes [25]. HA can also indirectly contribute to the viscosity of the synovial fluid and lubricate unloaded joints. Furthermore, since the pain-relieving effect of IA HA within the injected joint often persists for considerably longer than the half-life of HA, it is likely that it provides not only symptom-modifying but also disease-modifying effects. The half-life of unmodified HA solutions can be less than 1 day [26, 27]. Recently, a high molecular weight non-animal stabilized hyaluronic acid (NASHA) product) has gained popularity in the treatment of human arthritic conditions due to its long-acting pain relieving effects [8, 17, 18, 20–24].

Currently, only one double-blinded placebo-controlled clinical equine study on IA HA treatment has been published [15]. That study reported a superior effect of HA and polysulphated glycosaminoglycan compared with placebo in reducing the lameness score. Overall, the efficacy of intra-articular HA in relieving inflammation and pain has only been examined in a few clinical and experimental equine studies [11–15, 28, 29]. In horses with medically induced synovitis, White et al. [28] found HA to relieve lameness compared with placebo. However, in a surgically induced equine osteoarthritis model, no changes in the lameness score were detected after HA treatment compared with a control treatment [29].

Some osteoarthritis models in animals have shown that high molecular weight HA preparations are superior to less polymerized HA forms [30, 31]. In a clinical equine trial comparing HA preparations differing in molecular weight, better clinical results were reported using HA of a higher molecular weight [32], but more research on the different preparations is needed. In this study, a NASHA product for human use (Durolane, Bioventus) was used. It has a long half-life (up to 32 days) [33] and remains in the synovial structures for a considerably longer period than other HA products [17, 18, 34].

We compared an IA injection of this NASHA product with an IA injection of placebo. The aim of the study was to examine whether the change in lameness originating from a metacarpophalangeal joint, from before the injection to after the injection, would differ between the two treatment groups. The IA injections were given on the day of the clinical baseline examination and after two weeks the horses returned for a second clinical examination. Our hypothesis was that the outcome measures would improve more in the horses treated with NASHA compared with those treated with placebo.

## Methods

### Design

The study was carried out as a randomised double-blind and placebo-controlled trial with a parallel group design and equal allocation ratio. It was planned and it is reported according the CONSORT statement (http://www.consort-statement.org/checklists/view/32-consort/). The study was approved by the Viikki Campus Research Ethics Committee of the University of Helsinki (31 March 2010). All horse owners signed a study consent sheet before the start of the study and owner was allowed to stop the study of the horse without giving any particular reason. Reasons for removing the horse from the study could have been side-effects caused by the NASHA or placebo products, additional orthopaedic or other health problems occurring after the first clinical examination and/or owners’ lack of compliance or failure to follow the instructions given after the first clinical examination.

### Population

Horses with lameness due to synovitis of the metacarpo- or metatarsophalangeal joint, with or without mild osteoarthritis, were recruited to the study. Synovitis of all durations was acceptable. Only adult, non-geriatric (i.e. age between 4–17 years), Finnhorse, Standardbred and Warmblood horses of all disciplines were eligible. In addition, large-sized ponies (withers 140–148 cm) were accepted (for convenience, referred to as horses in the text). Inclusion criteria were a positive response to diagnostic intra-articular anaesthesia of the affected metacarpo- or metatarsophalangeal joint and no radiographic signs of remodelling of the affected joint. This excluded horses with more severe chronic osteoarthritis. In addition, horses with intra-articular osteochondral or other fragments were excluded. Bilaterally lame horses and horses that had received intra-articular medications such as corticosteroids or HA within the previous three months, or per oral NSAIDs within 15 days, were not eligible. Furthermore, horses with concurrent pathologies, such as clinically significant ligament, tendon or other soft tissue injuries in the affected limb, were excluded.

### Clinical exam and interventions

The study was advertised at stables, via the Internet page of the Faculty of the Veterinary Medicine [35], in equine clinics and in horse sport magazines. Horse owners contacted the University to have their horses included in the study and a telephone interview was conducted. An assistant not otherwise tied to the study enrolled the participants. Within a week after the horse owners had contacted the University to have their horses included, potential cases were presented to the Veterinary Teaching Hospital of the University of Helsinki for the first baseline clinical examination. This first examination was performed on the same day that each horse arrived at the hospital.

The horses were subjected to a complete lameness examination by the evaluating veterinarian (TMN). A standardized scale of 0–5 [36] was used to grade lameness. Effusion of the affected joint was recorded on a scale of 0–4 (0 = no effusion, 1 = mild, 2 = moderate, 3 = severe effusion, 4 = severe swelling of the joint region) [28, 37]. Other palpation findings, such as thickening of the joint capsule, were also recorded (yes/no thickening). A flexion test of the affected and the contralateral limb was performed and the lameness was recorded on a scale of 0–4 (0 = no increase, 1 = slight increase, 2 = moderate increase, 3 = considerable increase in the baseline lameness, 4 = non-weight-bearing lameness) [29]. To exclude confounding flexing reactions, for instance because of sensitiveness to handling, and thereby help the researchers to evaluate the reaction of the affected limb, the contralateral non-lame leg was always flexed first. The result of this contralateral flexion test was not recorded.

Pain when flexing the affected distal limb was also recorded on a scale of 0–3. This pain score was created by the authors and was recorded as follows: 0 = no pain on flexion, 1 = mild pain, i.e. the horse shows some reaction, such as moving the limb, 2 = moderate pain, i.e. the horse retracts the limb repeatedly during the 1 min flexion period, 3 = severe pain, i.e. the flexion test cannot be properly performed.

To localize the lesion and to decide if the horse was eligible for the study, a routine diagnostic aseptic arthrocentesis with an 18-gauge/3.8-cm needle was performed through the lateral sesamoidean ligament and 10 mL mepivacaine hydrochloride (Scandicain, Astra Zeneca) was injected into the joint. The response to the intra-articular anaesthesia was evaluated 10 min post-injection and was considered positive if a subjectively evaluated 80–100 % amelioration of lameness was evident. Radiographic examination of the joint with four standard views was performed (lateromedial, dorsopalmar, dorsolateral-palmaromedial and dorsomedial-palmarolateral), and all radiographs were evaluated by the same veterinarian (TMN).

Before starting the study, a non-blinded assisting technician created a computer generated randomization list using an Internet-based program [38]. The block size was 4 and no strata were used. Horses fulfilling the inclusion criteria were assigned to the treatment or control group according to the randomization list. The list and the NASHA and placebo products were kept in a locked locker at the university that only the non-blinded assisting veterinarian and his technician could access. Horses in the treatment group were injected with 3 mL NASHA into the affected joint, while horses in the control group received an equivalent volume of the sterile 0.9 % saline solution (Sodium chloride 9 mg/mL, B Braun) into the joint. Injections were administered on the same day that each horse arrived at the hospital, after confirming eligibility, the source of lameness and evaluating the radiographs.

Treatments were double-blinded so that the assisting veterinarian prepared the syringes according to the randomization list and performed the NASHA and saline injections, and neither the evaluating veterinarian (TMN) nor the owners were allowed in the treatment room during the procedure. Owners remained blinded until after the second clinical examination and the evaluating veterinarian (TMN) until after the statistical analyses were performed.

#### Second clinical examination and follow-up

Horses were allowed 30 min hand-walking per day and free access to a small paddock during the following two weeks, after which the second clinical examination was performed. Palpation findings and other outcome measures were re-evaluated and recorded (TMN). After the second clinical examination, the double-blinded trial part was concluded and the horses in the control group also received an IA NASHA injection to treat the lameness. To ensure that all treatment injections were performed by the same veterinarian, the assisting veterinarian also injected the placebo group with the NASHA. Follow-up information was collected two and a half to three months post-treatment by interviewing all owners by telephone.

The primary outcome measure was the change in the lameness score from baseline to the second clinical examination in the two intervention groups. The secondary outcome measures were the change in the effusion of the affected joint, in the lameness after the flexion test and in pain in flexion from baseline to the second clinical examination.

#### Statistical methods

A sample size calculator [39] was used with a 95 % confidence level and 80 % power, and the sample size was estimated at 11–14 horses per group based on 87 % of cases showing clinical improvement in an earlier study on HA for the treatment of naturally occurring arthritic conditions in horses [13]. In the placebo group, the proportion that would improve was estimated to be 20–30 %, where 20 % is 10 % lower than the percentage that has been used in the placebo groups in human studies [40, 41].

Data analysis was performed with a computer-based statistical program (SPSS Software, IBM). The final values for the outcome measures were subtracted from the baseline values to form variables of change, allowing positive and negative values. These variables of change were used in the comparison between groups with the independent-samples Mann–Whitney *U*-test (ranks). The demographic data and outcome measures at baseline were compared between the two treatment groups using the independent-samples Mann–Whitney *U*-test, or in the case of nominal categorical variables, Fisher’s exact test or the likelihood ratio test. Furthermore, the outcome measures within groups were compared between the baseline examination and the second clinical examination using the Wilcoxon signed-rank test. All the above statistics were calculated for the population intended to treat (*n =* 30) and for the metacarpophalangeal patients only (*n =* 27). Value of *P <* 0.05 was considered significant.

## Results

**Fig. 1:**
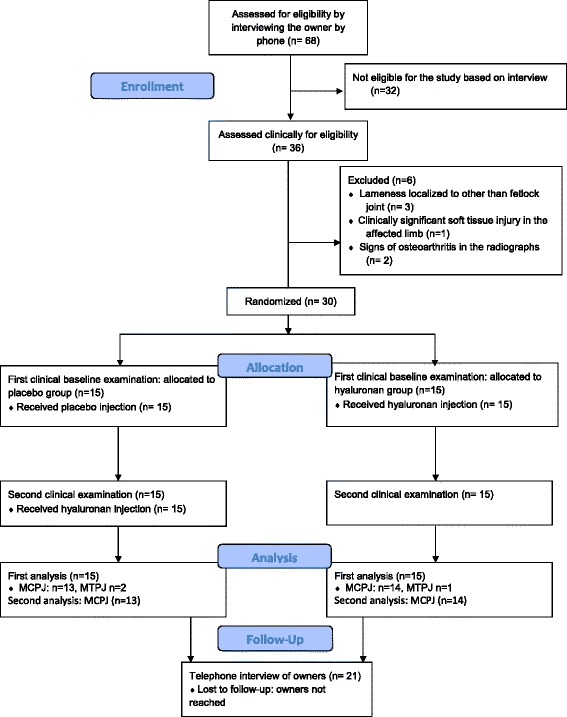
Flow diagram showing the number of horses in different phases of the study. MCPJ = metacarpophalangeal joint; MTPJ = metatarsophalangeal joint

Sixty-eight horse owners were interviewed, which resulted in 36 horses being invited to the first clinical baseline examination. Altogether, 30 horses fulfilling the inclusion criteria were included in the study. However, as only three horses that concluded the study had lameness due to synovitis of the metatarsophalangeal joint, these were omitted from some of the analyses, leaving 27 horses with synovitis of the metacarpophalangeal joint as the primary study population (Table 1, Fig. 1). All the 27 horses completed the study. For the included horses, all examinations were performed between May 2010 and May 2012.

**Table 1:** Demographic variables and outcome measures in the first clinical baseline examination of the placebo and NASHA groups (*n =* 27) and *P*-values for the comparison between treatment groups at the first clinical baseline examination

Variable	Placebo	NASHA	*P*-value
Number of horses	13	14	
Age (~years): Mean (range)	8.4 (4–17)	7.2 (4–12)	0.55
Gender: Mare/Stallion/Gelding %	46 %/15 %/39 %	43 %/21 %/36 %	0.09
Purpose: Harness race horse/Riding horse %	54 %/46 %	71 %/29 %	0.44
Breed: Standardbred/Finnhorse/Wamblood/Pony %	31 %/39 %/15 %/15 %	43 %/50 %/7 %/0 %	0.65
Affected limb: RF/LF %	46 %/54 %	71 %/29 %	0.25
Baseline lameness (AAEP)^a^: 0/1/2/3/4/5 %	0 %/15 %/39 %/46 %/0 %/0 %	0 %/36 %/57 %/7 %/0 %/0 %	0.05
Effusion^b^: 0/1/2/3/4 %	23 %/46 %/0 %/31 %/0 %	29 %/29 %/35 %/7 %/0 %	0.83
Lameness in the flexion test^c^: 0/1/2/3/4 %	0 %/0 %/8 %/61 %/31 %	0 %/0 %/29 %/50 %/21 %	0.33
Pain in flexion^d^: 0/1/2/3 %	0 %/31 %/23 %/46 %	7 %/29 %/36 %/28 %	0.46
Mild findings in radiographs: Yes/No %	54 %/46 %	50 %/50 %	1.00

^a^ AAEP scale 0–5

^b^ 0 = no effusion, 1 = mild, 2 = moderate, 3 = severe effusion, 4 = severe swelling of the metacarpal joint region

^c^ 0 = no increase, 1 = slight increase, 2 = moderate increase, 3 = considerable increase in baseline lameness, 4 = non-weight-bearing lameness

^d^ 0 = no pain at flexion, 1 = mild pain, 2 = moderate pain, 3 = severe pain

In 16 horses, lameness had previously been localised to the joint from which the lameness originated at baseline. Fifteen horses had received HA and/or corticosteroid treatment more than 3 months before entering the study in the same joint from which the lameness originated at baseline, while nine horses had not previously received any medications for musculoskeletal problems.

No significant differences in signalment, the use of the horse, outcome measures or the number of horses with findings in radiographs were found between the NASHA and placebo treatment groups at the first clinical (baseline) examination (Table 1). The baseline lameness score ranged from 1 to 3 (out of 0–5) and the most frequent lameness score was 2 (*n =* 13, 48 %). In 7 (26 %) horses, the lameness score was 1 and in 7 (26 %) cases 3.

When the change in the lameness score as the primary outcome measure was compared between the NASHA and the placebo groups, no significant difference was seen (*P =* 0.94, Table 2). When the changes in the secondary outcome measures were compared respectively, a significant difference was recorded for the change in the response to the flexion test (*P =* 0.01, Table 2). In contrast, the change in effusion and in the pain in flexion was similar in the placebo and NASHA groups (*P =* 0.94 and *P =* 0.27, respectively, Table 2). All results were similar when the three horses with metatarsophalangeal joint problems had been included.

**Table 2:** Medians (and ranges) of outcome measures in the placebo and NASHA groups, with *P*-values for the within-groups comparison of outcome measures between the first baseline and second clinical (end) examination, and *P*-values for the comparison between groups (*P* of change between interventions) of the change in outcome measures after the second clinical examination

Outcome measure	Placebo baseline	Placebo end	*P* within placebo	NASHA baseline	NASHA end	*P* within NASHA	*P* for change between interventions
Lameness^a^	2 (1–3)	2 (0–3)	0.005	2 (1–3)	0 (0–3)	0.02	0.94
Effusion^b^	1 (0–3)	1 (0–3)	0.48	1 (0–3)	1 (0–3)	0.56	0.94
Flexion test^c^	3 (2–4)	3 (1–4)	0.07	3 (2–4)	1 (0–3)	0.002	0.01
Pain^d^	2 (1–3)	1 (0–3)	0.04	2 (0–3)	0 (0–2)	0.01	0.27

^a^ AAEP scale 0–5

^b^ 0 = no effusion, 1 = mild, 2 = moderate, 3 = severe effusion, 4 = severe swelling of the metacarpal joint region

^c^ 0 = no increase,1 = slight increase, 2 = moderate increase, 3 = considerable increase in baseline lameness, 4 = non-weight-bearing lameness

^d^ 0 = no pain at flexion, 1 = mild pain, 2 = moderate pain, 3 = severe pain

No adverse effects of either intra-articular NASHA or saline injection could be seen in any of the horses. Based on the follow-up telephone interview at 2.5–3 months, 14 of the 21 horses (67 %) had been able to return to their previous level of use, but two of these had later developed other musculoskeletal problems. Six horse owners were not reached for the follow-up interview. Seven of the 21 horses (33 %) were no longer used in the same discipline or at the same performance level.

## Discussion

In this study, the effect of intra-articular NASHA in relieving synovitis and lameness in clinical patients was evaluated. Apart from the study of Gaustad and Larsen [15], this is to our knowledge the only double-blinded placebo-controlled clinical equine study on intra-articular HA, and this study focused on a single joint. Although the difference in the baseline lameness score was almost statistically significant, the groups of the present study were comparable (Table 1), as the baseline clinical and demographic variables did not significantly differ between them. This is important in a clinical study, since variables such as the level of physical activity of the horses cannot be fully controlled for. Gaustad and Larsen [15] also reported comparable treatment groups in their study (*n =* 77), but the HA treatment group (*n =* 28) was not separated from the PSGAG group (*n =* 27). Moreover, their study included horses with traumatic arthritis as well as other diagnoses in several high motion joints but the distribution of diagnoses between groups was not reported.

Most of the previous studies on IA HA treatment in horses are not comparable with the present study, since only two of them [15, 28] were blinded placebo-controlled studies and only one of the clinical studies [14] focused on a single joint. Furthermore, White et al. [28] used a prospective experimental study design with only 4 horses per group. Lameness was evaluated by measuring stride length, and not using the more subjective but, standardised, AAEP scale [36], which also takes into account the weight-bearing component of lameness. As in the present study, Gaustad and Larsen [15] found a significantly reduced lameness score in both the treatment and placebo groups. However, in contrast to their study, improvement in the lameness score of horses injected IA with NASHA in the present study, was not better than in the placebo group. The different result in the study of Gaustad and Larsen [15] might be explained by the more severe mean baseline lameness, the larger sample size, the multiple joints involved, or the presence of different joint diseases, compared with the present study. As in our study, Frisbie et al. [29] reported a lack of improvement in the lameness score with respect to treatment when HA was administered to horses with experimentally induced osteoarthritis. However, histologically, significantly less cartilage fibrillation was seen with HA treatment compared with controls [29].

A response to the flexion test was the only outcome measure which improved more in the NASHA group compared with the placebo group (Table 2). In addition to the lameness score, the flexion pain score improved both within the placebo and the NASHA groups (Table 2). It is possible that saline by itself might have had a positive effect, as also shown by Gaustad et al. [42]. They reported a significant improvement in horses with arthritis after repeated injections of a 2 mL NaCl solution. There was also a significant decrease in the lameness score when this NaCl-injected group was compared with the rest-only group. The synovial membrane has been shown to respond to IA injections of saline, and an increase in the synovial fluid HA concentration after an injection of saline into equine joints has been reported [43]. Furthermore, in both the study of Gaustad et al. [42] and the present study, the therapeutic effect of the local anaesthetic cannot be excluded. However, the improvement also seen in the placebo group might be explained by the two weeks of light exercise (half an hour of hand-walking per day) between clinical examinations, which may have been sufficient to improve relatively low-grade lameness, especially in harness racehorses undergoing heavy training. Furthermore, our study design was based on the assumption of a large difference in lameness improvement between groups based on an earlier study of HA for the treatment of naturally occurring arthritic condition in horses [13], so our originally calculated number of cases was probably too low to show smaller differences between groups.

Despite the lack of improvement in the other outcome measures of the present study, the greater improvement in the flexion test score in the NASHA group (Table 2) shows that IA NASHA may have some beneficial clinical sign modifying effects. Flexion tests are an integral part of a lameness examination. Although flexion tests may have some limitations, the distal limb flexion test has been shown to be very specific for the disease and the pain arising from the metacarpophalangeal joint [44].

Treatment with high molecular weight IA HA is of interest in human orthopaedics, and recent research has demonstrated it to be effective and long-acting in the treatment of human osteoarthritis [20–24]. Based on cross-linking, the NASHA has a prolonged residence time and results in a viscous HA gel in the joint [34]. Previously, exogenous HA has been shown to disappear from the joint within seven days [45], and synovitis also enhances the clearance of HA from the joint [46]. However, Altman et al. [18] found a prolonged reduction in pain after a NASHA treatment in human knee osteoarthritis patients with a maximum effect at 6 weeks post-treatment. Similarly, Gaustad and Larsen [15] reported that a significant number of horses became sound between 3 and 6 weeks post-injection. Exogenous HA has also been shown to increase the synthesis of HA by human synovial cell lines, which could explain the prolonged effect of exogenous HA [47]. Thus, the improvement in lameness in the NASHA group might have been greater if the period between the examinations in the present study had been longer than 2 weeks. However, since our study population consisted of clinical client-owned patients, it would have been unethical to delay the treatments of the horses receiving placebo for a longer period and all the first placebo-injected horses were therefore treated at the second examination. The third assessment was performed as a telephone interview to avoid long-distance travel for many of the patients. Most horses (14 of 21, i.e. 67 %) were able to perform at their previous level based on subjective assessment by owners or trainers.

A clinical study design sets limits on a placebo-control set-up. In addition, the lack of significant difference in the change of lameness score between the NASHA and placebo groups also reflects the difficulty in conducting clinical studies, as variation between intervention groups is always inevitably present, despite the lack of a statistically significant difference in demographic variables and in baseline outcome measures, as in the present study (Table 1). The severity of the disease can vary, despite a uniform lameness score or other clinical scores.

Selection bias was minimized by recruiting horses through several media and from a variety of riding and racehorse stables around southern Finland. On the other hand, this may also have caused a selection of horses which have been unresponsive to previous treatments or a selection of horse owners or trainers who overall train their horses differently compared with the general horse owner or trainer population. The population in the present study may also have been too heterogeneous, i.e. with variety of disciplines, training levels and training conditions may have caused excessive variation in the response to the NASHA injection and in the lack of a significant difference in most of the outcome measures.

No adverse effects of the intra-articular NASHA injection could be seen in any of the horses. A transient treatment related adverse effect of IA HA has been reported in up to 10.0 % of cases in an equine study [13] and 12.5 % in a human NASHA study [18].

## Conclusions

In the present study, a single IA NASHA injection was not better than IA saline for reducing lameness in horses with synovitis or mild osteoarthritis in the metacarpophalangeal joint. However, this study shows indication that it may have some beneficial effects in modifying clinical signs. A single IA injection of NASHA might be useful in the treatment of acute synovitis, but more research is needed on the effects of NASHA in other equine joint disease states as well as on its mechanism of action.

## Abbreviations

HA: hyaluronic acid
IA: Intra-articular
NASHA: non-animal stabilized hyaluronic acid
